# Three new species of the genus *Aporcelaimoides* Heyns, 1965 from Vietnam (Nematoda, Dorylaimida, Aporcelaimidae), with an updated taxonomy of the genus

**DOI:** 10.3897/zookeys.516.10087

**Published:** 2015-08-06

**Authors:** Sergio Álvarez-Ortega, Thi Anh Duong Nguyen, Joaquín Abolafia, Thi Thanh Tam Vu, Reyes Peña-Santiago

**Affiliations:** 1 Departamento de Biología Animal, Biología Vegetal y Ecología, Universidad de Jaén, Campus ‘Las Lagunillas’ s/n, Edificio B3, 23071– Jaén, Spain; 2 Department of Terrestrial Ecology, Zoological Institute, University of Cologne, Zülpicher Straße 47b, D-50674 Cologne, Germany; 3 Institute of Ecology and Biological Resources, Vietnam Academy of Science and Technology, 18 Hoang Quoc Viet, Hanoi, Vietnam

**Keywords:** Description, morphology, morphometrics, new combinations, new species, taxonomy, *Sectonema*, SEM

## Abstract

Three new species of *Aporcelaimoides* from natural habitats in Vietnam are studied, described and illustrated, including line drawings, LM and/or SEM pictures. *Aporcelaimoides brevistylum* **sp. n.** is characterized by its body 1.95–2.90 mm long, lip region offset by deep constriction and 17–18 µm broad, ventral side of mural odontostyle 11–14 µm long with aperture occupying 62–71% of its length, neck 663–767 µm long, pharyngeal expansion occupying 58–66% of total neck length, uterus a simple tube 85–182 µm long, *pars refringens vaginae* absent, *V* = 55–63, tail short and rounded (34–46 µm, *c* = 49–76, *c*’ = 0.6–0.8), spicules 67–86 µm long, and one ventromedian supplement out the range of spicules. *Aporcelaimoides minor* **sp. n.** is distinguished in having body 2.09–2.61 mm long, lip region offset by deep constriction and 19–20 µm broad, mural odontostyle 14–16 µm long at its ventral side with aperture occupying 73–84% of its length, neck 579–649 µm long, pharyngeal expansion occupying 57–66% of total neck length, uterus a simple tube 44–69 µm long, *pars refringens vaginae* well developed, *V* = 48–56, female tail very short, rounded conoid or truncate (14–26 µm, *c* = 90–146, *c*’ = 0.3–0.6), and male unknown. *Aporcelaimoides silvaticum* **sp. n.** is characterized by its body 2.09–2.60 mm long, lip region offset by depression and 17–18 µm broad, mural odontostyle 11–12 µm long at its ventral side with aperture occupying 60–66% of its length, neck 597–720 µm long, pharyngeal expansion occupying 58–64% of total neck length, uterus a simple tube 128–243 µm long, *pars refringens vaginae* well developed, *V* = 58–60, tail short and rounded (27–37 µm, *c* = 67–94, *c*’ = 0.6–0.7), spicules 64–75 µm long, and two or three widely spaced ventromedian supplements bearing hiatus. The genus *Aporcelaimoides* is restored, its diagnosis emended, and three species of *Sectonema*, namely *Sectonema amazonicum*, *Sectonema haguei* and *Sectonema moderatum*, transferred to it. An updated list of its species, a key to their identification and a tabular compendium with the most important morphometric features are also presented.

## Introduction

The genus *Aporcelaimoides* is an interesting aporcelaimoid taxon, created by Heyns (1965) to accommodate two new species, namely *Aporcelaimoides probulbum* (type species) and *Aporcelaimoides californicum*. It was originally characterized among other features by its “Spear dorylaimoid, with a large dorsal aperture, but the basal part of the spear much narrower than the lumen of the pharynx, and situated ventrally in the pharynx”. Later, Siddiqi (1995) regarded it as a junior synonym of *Sectonema* Thorne, 1930, an action that, several years later, was followed by Andrássy (2009). However, this taxonomical decision deserves further analyses since important morphological differences exist between both genera, being especially important those affecting the nature of the stomatal protrusible structure.

The study of dorylaimid fauna from Vietnam has received poor attention. Several authors (Vu et al. 2010; Nguyen et al. 2011; Gagarin and Gusakov 2012 and 2013a,b; Nguyen et al. 2014) discovered some new species and reported for the first time information about other known species. It is suggested that Vietnamese dorylaimid fauna might be highly diverse. This is the first contribution in a series devoted to study the aporcelaimid fauna of this Asian country.

During a general nematological survey conducted during the last five years to study the diversity of the Vietnamese nematode fauna, several specimens of the genus *Aporcelaimoides* were collected from natural areas in Vietnam. Their detailed examination revealed they belonged to three unknown forms, which are herein described. Besides, the study of this nematode material has confirmed relevant data to reconsider the identity of the genus *Aporcelaimoides*.

## Material and methods

### Nematodes

Nematodes were collected from several natural areas in Vietnam, extracted from soil samples using the methods of Baermann (1917) and Flegg (1967) somewhat modified, relaxed and killed by heat, fixed in 4% formaldehyde, and processed to anhydrous glycerine following Siddiqi’s (1964) technique. Finally, the specimens were mounted on permanent glass slides to allow handling and observation under LM.

### Light microscopy

Nematodes were measured using a light microscope. Morphometrics included de Man’s indices and most of the usual measurements. The location of the pharyngeal gland nuclei is expressed according to Loof and Coomans (1970) and spicule terminology follows Peña-Santiago et al. (2014). Some of the best preserved specimens were photographed with a Nikon Eclipse 80i microscope and a Nikon DS digital camera. Raw photographs were edited using Adobe® Photoshop® CS. Drawings were made using a *camera lucida*.

### Scanning electron microscopy

After their examination and identification, a few specimens preserved in glycerin were recycled to their observation under SEM following the protocol by Abolafia and Peña-Santiago (2005). The nematodes were hydrated in distilled water, dehydrated in a graded ethanol and acetone series, critical point dried, coated with gold, and observed with a Zeiss Merlin microscope.

## Taxonomy

### Aporcelaimoides brevistylum sp. n.

Taxon classificationAnimaliaDorylaimidaAporcelaimidae

http://zoobank.org/0D05FA79-C8FB-46A8-9496-02F6F9D8E6E7

**Figure 1.**
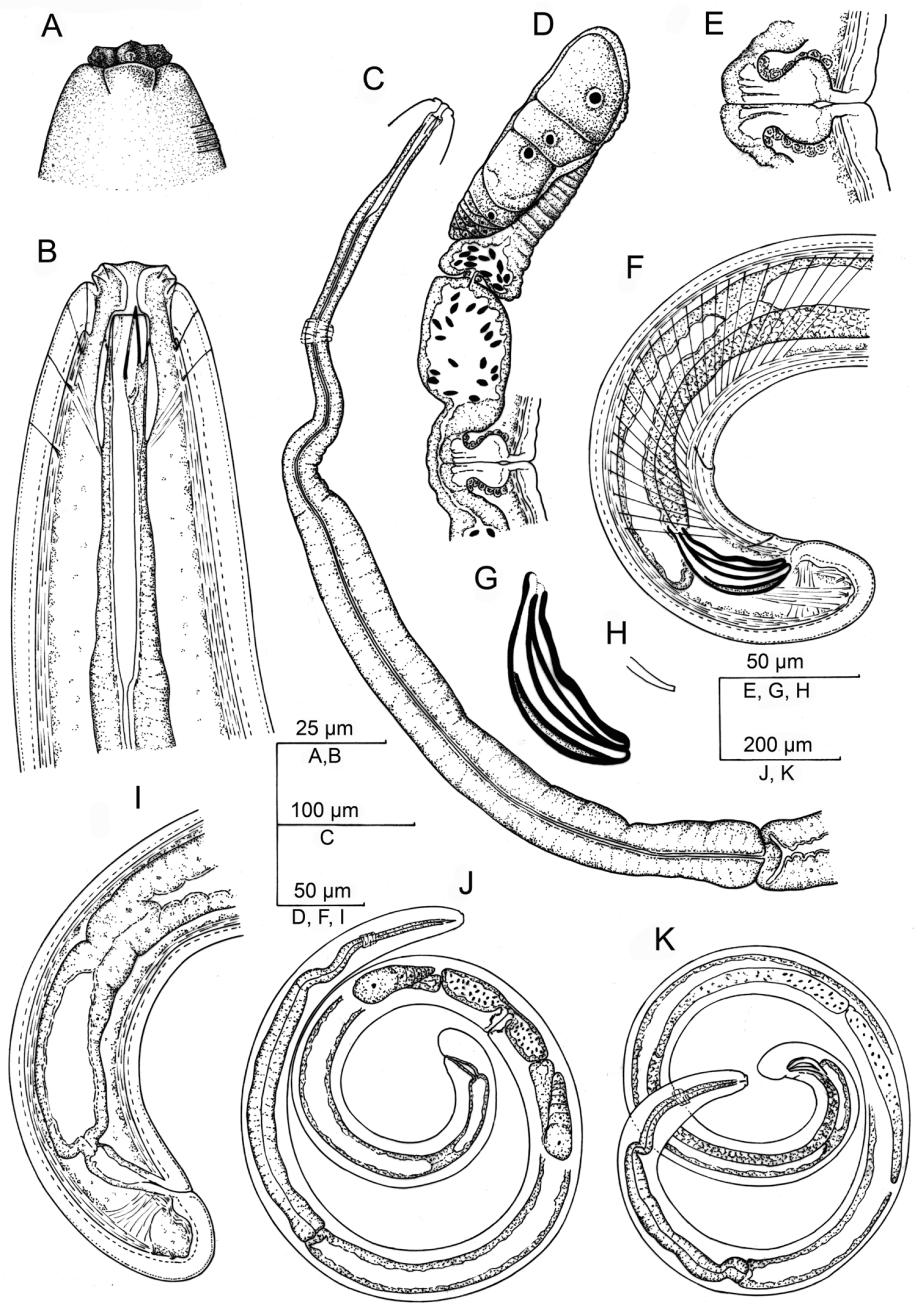
*Aporcelaimoides brevistylum* sp. n. (Line drawing). **A** Lip region in surface, lateral view **B** Anterior region in median lateral view **C** Neck **D** Female, anterior genital branch and vagina **E** Vagina **F** Male, posterior body region **G** Spicule **H** Lateral guiding piece **I** Female, posterior body region **J** Female, entire **K** Male, entire.

**Figure 2.**
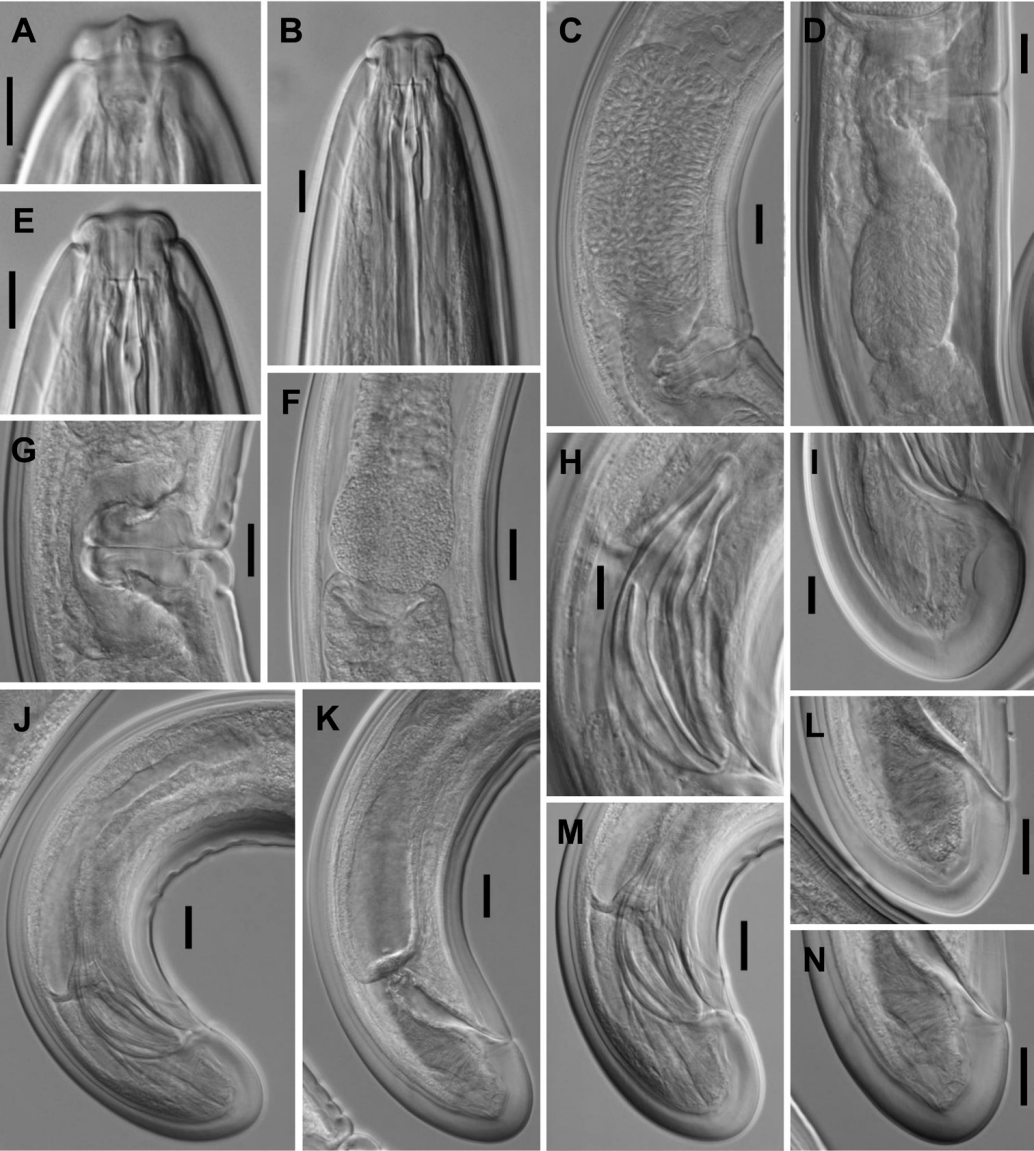
*Aporcelaimoides brevistylum* sp. n. (LM, type population). **A** Anterior region in surface, lateral view **B, E** Anterior region in median, lateral view **C, D** Uterus, containing sperm cells inside **F** Pharyngo-intestinal junction **G** Vagina **H** Spicule **I, M** Male, caudal region **J** Male, posterior body region **K** Female, posterior body region **L, N** Female, caudal region. Scale bars: 10 µm (**A, B, E, H, I**); 20 µm (**C, D, F, G, J–N**).

**Figure 3.**
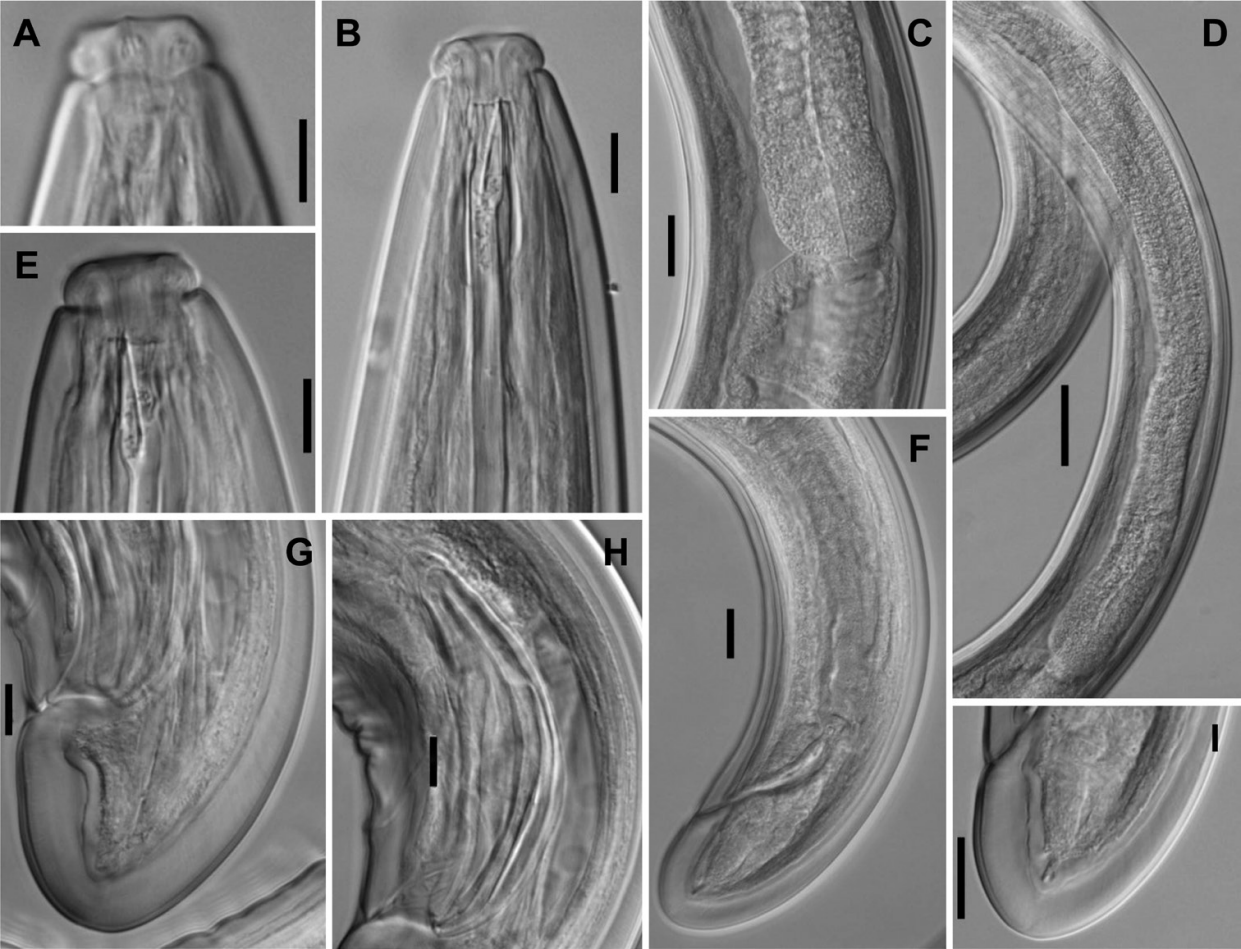
*Aporcelaimoides brevistylum* sp. n. (LM, other population). **A** Anterior region in surface, lateral view **B, E** Anterior region in median, lateral view **C** Pharyngo-intestinal junction **D** Pharyngeal expansion **F** Female, posterior body region **G** Male, caudal region **H** Spicules **I** Female, caudal region. (Scale bars: 10 µm (**A, B, E, G, H**); 20 µm (**C, F, I**); 50 µm (**D**).

**Figure 4.**
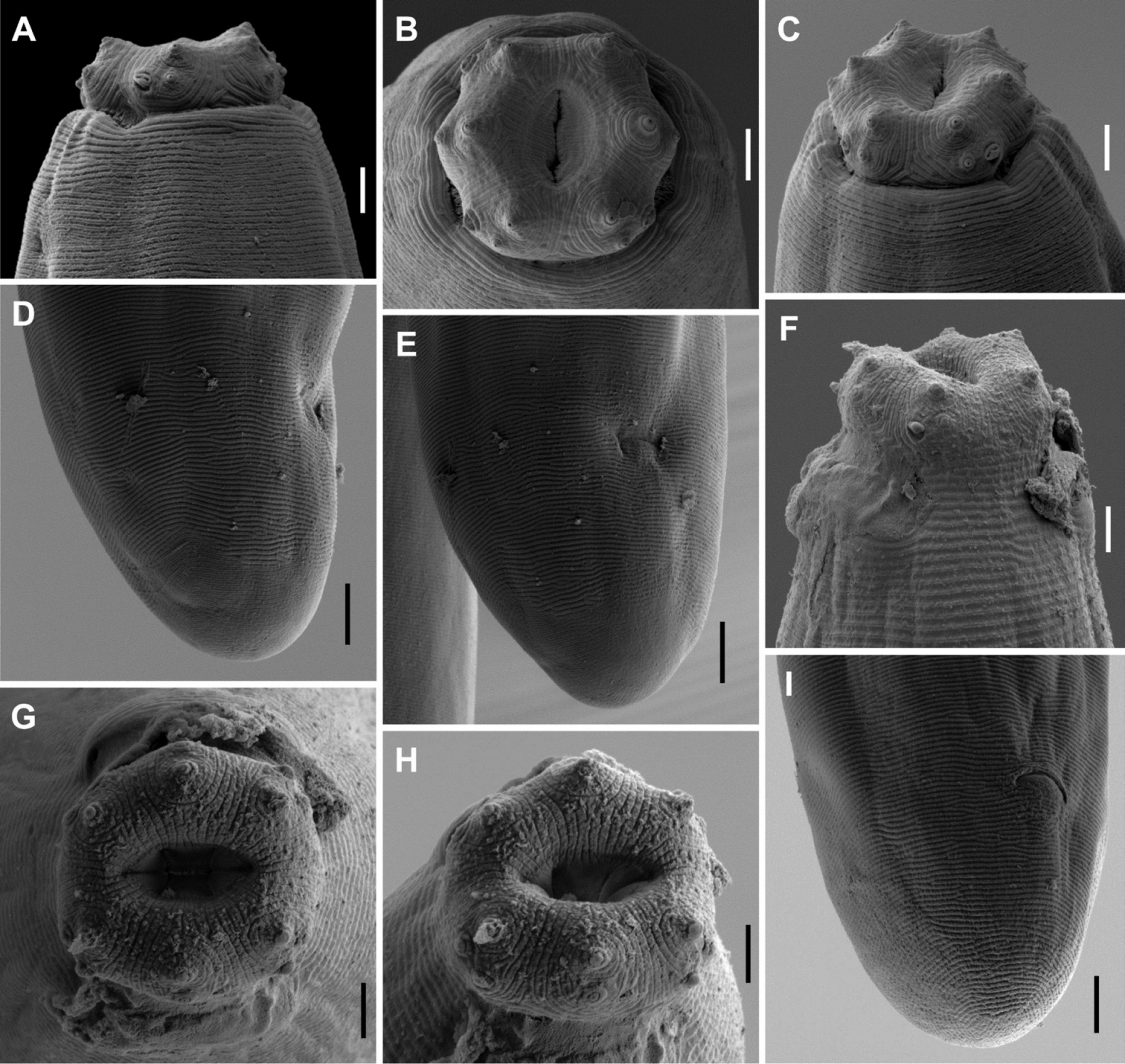
*Aporcelaimoides brevistylum* sp. n. **A–E** and *Aporcelaimoides silvaticum* sp. n. **F–I** (SEM, juvenile). **A, C, F** Lip region in ventral view **B, G, H** Lip region in face view **D, E, I** Caudal region in lateral (**D**) or subventral (**E, F**) view. Scale bars: 2 µm (**A–C, F–H**); 5 µm (**D, E, I**).

Figs 1 , 2 , 3 , 4A–E

#### Material examined.

Twelve females and fourteen males from two localities, in variable state of preservation.

#### Measurements.

See Table 1.

**Table 1.** Morphometrics of *Aporcelaimoides brevistylum* sp. n. Measurements in µm (except L, in mm), and in the form: mean ± standard deviation (range).

Population		Chu Yang Sin National Park			Bidoup-Nui Ba National Park		Total range	
		Holotype	Paratypes					
Character	**n**	♀	8♀♀	10♂♂	3♀♀	4♂♂	12♀♀	14♂♂
L		2.55	2.59 ± 0.16 (2.33–2.77)	2.24 ± 0.18 (1.95–2.60)	2.77 ± 0.15 (2.60–2.90)	2.44 ± 0.03 (2.41–2.49)	2.33–2.90	1.95–2.60
a		30	29.2 ± 3.2 (25–33)	29.0 ± 1.7 (27–32)	28.4 ± 3.4 (26–32)	31.8 ± 2.9 (29–35)	25–33	27–35
b		3.3	3.4 (n=1)	3.3 (n=1)	3.5 (n=1)	3.6 ± 0.1 (3.6–3.7)	3.3–3.5	3.3–3.7
c		69	67.8 ± 7.2 (58–76)	59.2 ± 5.7 (49–67)	70.6 ± 4.0 (67–75)	64.8 ± 4.0 (61–69)	58–76	49–69
c’		0.6	0.7 ± 0.1 (0.6–0.8)	0.7 ± 0.1 (0.6–0.8)	0.7 ± 0.1 (0.7–0.8)	0.7 ± 0.0 (0.7–0.8)	0.6–0.8	0.6–0.8
V		61	59.0 ± 2.3 (55–63)	-	60.0 ± 1.0 (59–61)	-	55–63	-
Lip region diam.		18	17.4 ± 0.5 (17–18)	16.8 ± 0.2 (17–17)	17.0 ± 0.5 (17–18)	17.5 ± 0.0 (18–18)	17–18	17–18
Mural odontostyle length at ventral side		13	12.0 ± 0.6 (11–13)	11.8 ± 0.3 (11–12)	12.9 ± 0.7 (13–14)	11.4 ± 0.3 (11–12)	11–14	11–12
Mural odontostyle length at dorsal side		15	13.9 ± 0.5 (13–14)	13.5 ± 0.4 (13–14)	15.2 ± 1.2 (14–17)	13.4 ± 0.2 (13–13)	13–17	13–14
Odontophore length		49	47.8 ± 2.1 (44–50)	45.7 ± 2.0 (43–49)	48.4 ± 1.8 (46–50)	45.9 ± 1.1 (45–48)	44–50	43–49
Guiding ring from ant. end		11	12.1 ± 0.3 (12–13)	11.0 ± 0.7 (10–12)	12.5 ± 0.3 (12–13)	11.3 ± 0.2 (11–12)	11–13	10–12
Neck length		767	695 (n=1)	663 (n=1)	740 (n=1)	677 ± 11 (664–685)	695–767	663–685
Pharyngeal expansion length		508	405 (n=1)	389 (n=1)	458 (n=1)	402 ± 13 (387–412)	405–508	387–412
Diam. at neck base		71	81.5 ± 9.5 (70–96)	72.6 ± 9.9 (56–85)	81, 88 (n=2)	74.7 ± 2.5 (73–77)	70–96	56–85
at midbody		85	89.2 ± 10.8 (72–106)	77.4 ± 7.1 (66–88)	99.0 ± 16.0 (81–109)	77.4 ± 6.9 (69–84)	72–109	66–88
at anus		57	58.0 ± 3.0 (54–63)	53.7 ± 2.7 (49–58)	53.3 ± 2.0 (52–56)	51.7 ± 2.0 (49–53)	52–63	49–58
Prerectum length		134	128.1 ± 15.7 (111–149)	177 ± 27 (149–218)	131 (n=1)	199 ± 33 (166–232)	111–149	149–232
Rectum/cloaca length		63	61.1 ± 3.9 (55–65)	62.7 ± 5.3 (56–70)	62.1 ± 1.0 (61–63)	62.7 ± 1.9 (61–65)	55–65	56–70
Tail length		37	38.9 ± 3.5 (35–46)	38.0 ± 2.7 (34–42)	39.3 ± 2.1 (38–42)	37.9 ± 1.9 (36–40)	35–46	34–42
Spicule length		-	-	79.6 ± 5.3 (67–86)	-	82.0 ± 2.9 (80–85)	-	67–86
Ventromedian supplements		-	-	1.0 ± 0.0 (1–1)	-	1.0 ± 0.0 (1–1)	-	1–1

#### Description.

*Adult*. Moderately slender to slender nematodes of medium size, 1.95–2.90 mm long. Body cylindrical, distinctly tapering towards the anterior end, less so towards the posterior one because the caudal region is rounded. Habitus regularly (often strongly) curved ventrad after fixation, usually spiral-shaped. Cuticle three-layered, especially distinguishable at caudal region, where it consists of thinner outer layer bearing very fine transverse striation through the entire body, thicker intermediate layer with radial striation and thin inner layer; thickness 3–5 µm at anterior region, 4–7 µm in mid-body and 9.0–12.5 µm on tail. Lateral chord 8–20 µm wide at mid-body, occupying one-eighth to less than one-fifth (12–18%) of mid-body diameter. Three ventral and three dorsal body pores are usually present at level of mural odontostyle-odontophore, their corresponding ducts appearing especially thickened beneath intermediate cuticle layer. Lip region offset by deep constriction, 2.7–3.3 times as wide as high and one-fifth to two-sevenths (18–30%) of body diameter at neck base; lips (under SEM) amalgamated; labial papillae button-like, very perceptible and protruding under LM, surrounded by a ring-like annulus (occasionally two annuli), the inner ones at the margin of oral field; cephalic papillae larger than the labial ones, with an oval transverse slit; oral aperture a dorso-ventral, slightly hexagonal orifice, the lip region hence showing a biradial symmetry. Amphid fovea cup-shaped, its opening occupying 9–11 µm or one-half to two-thirds (52–64%) of lip region diameter. Cheilostom nearly cylindrical, lacking any differentiation. Mural odontostyle attached subventrally and comparatively short, 4.1–5.4 times as long as wide, 0.6–0.8 times as long as lip region diameter, and 0.43–0.61% of body length; aperture 8–9 µm long or up to five-sevenths (62–71%) its length. Guiding ring simple, somewhat plicate, at 0.6–0.8 lip region diameters from anterior end. Odontophore linear, rod-like, 3.4–4.2 times the mural odontostyle length. Anterior region of pharynx enlarging very gradually; basal expansion 9.5–12.6 times as long as wide, 4.6–7.2 times as long as body diameter, and occupying 58–66% of total neck length; gland nuclei obscure in most specimens examined, DN = 50 (n=1) and S_2_N = 84 (n=1). Nerve ring located at 154–185 µm from anterior end or 21–26% of total neck length. Cardia rounded conoid, 10–14 × 14–18 µm; a ring-like structure is present surrounding its junction to pharyngeal base. Tail short and rounded; inner core with irregular shape at tail end. Caudal pores two pairs, one lateral, another sub-lateral.

*Female*. Genital system didelphic-amphidelphic, with both branches almost equally and well developed, the anterior 207–254 µm long or 9–10% of body length and the posterior 233–300 µm long or 9–13% of body length. Ovaries moderately sized, usually not surpassing the sphincter level, the anterior 95–365 µm, the posterior 106–316 µm long; oocytes arranged first in two or more rows, then in a single row. Oviduct 96–124 µm long or 1.0–1.4 times the corresponding body diameter, and consisting of a slender part with prismatic cells and a well developed *pars dilatata* bearing wide lumen that often containing sperm cells inside. Oviduct-uterus junction marked by a sphincter. Uterus a short, simple, tube-like structure 85–182 µm long or 1.0–2.1 times the corresponding body diameter, most specimens with abundant sperm cells inside. Uterine eggs ovoid, 153 (n=1) × 79, 85 (n=2) µm, 1.8 (n=1) times as long as wide. Vagina extending inwards 43–57 µm or four-ninths to two-thirds (45–65%) of body diameter: *pars proximalis* 32–44 × 28–34 µm, with somewhat sigmoid walls and surrounded by weak musculature; *pars refringens* absent; and *pars distalis* well developed, 11–14 µm long. Vulva a post-equatorial transverse slit. Prerectum 1.8–2.6, rectum 1.0–1.2 anal body diameters long.

*Male*. Genital system diorchic, with opposite testes. In addition to the ad-cloacal pair, situated at 15–20 µm from cloacal aperture, there is only one ventromedian supplement located out the range of spicules, at 48, 58 (n=2) µm from ad-cloacal pair. Spicules distinctly robust and massive, especially in its posterior half, 3.4–4.6 times its maximum width, 1.2–1.7 times the body diameter at level of the cloacal aperture: dorsal contour regularly convex, ventral contour very weakly concave, with shallow or weak hump and hollow; curvature 126–142°; head occupying 7–21% of spicule total length, its dorsal contour conspicuously curved at its anterior end and longer than the ventral one, which is short and straight; median piece 7.2–10.9 times as long as wide, occupying 35–50% of spicule maximum width, reaching the posterior tip; posterior end 5–9 µm wide. Lateral guiding pieces 13–17 µm long, 3.5–5.1 times as long as wide. Prerectum 2.9–4.4, cloaca 1.1–1.3 the corresponding body widths long.

#### Diagnosis.

The new species is characterized by its body 1.95–2.90 mm long, lip region offset by deep constriction and 17–18 µm broad, ventral side of mural odontostyle 11–14 µm with aperture occupying 62–71% of its length, neck 663–767 µm long, pharyngeal expansion 387–508 µm long or occupying 58–66% of total neck length, uterus a simple tube and 85–182 µm long or 1.0–2.1 times the corresponding body diameter, *pars refringens vaginae* absent, *V* = 55–63, female tail short and rounded (35–46 µm, *c* = 58–76, *c*’ = 0.6–0.8), male tail similar to that of female (34–42 µm, *c* = 49–69, *c*’ = 0.6–0.8), spicules 67–86 µm long, and one ventromedian supplement bearing hiatus.

#### Relationships.

In having short mural odontostyle (11–14 µm at its ventral side) and *pars refringens vaginae* absent, the new species is morphologically close to *Aporcelaimoides californicum* Heyns, 1965 and *Aporcelaimoides probulbum* Heyns, 1965, but it can be distinguished from both species in its smaller (*L* = 1.95–2.90 *vs* *L* = more than 3) and less slender (*a* = 25–35 *vs* *a* ≥ 41) body. Besides, *Aporcelaimoides brevistylum* sp. n. differs from *Aporcelaimoides californicum* in its comparatively longer neck (*b* = 3.3–3.7 *vs* *b* = 7.6), larger mural odontostyle aperture (occupying 62–71% *vs* one-half of its length), more posterior vulva (*V* = 55–63 *vs* *V* = 51), shorter uterus (85–182 µm or 1.0–2.1 times the corresponding body diameter *vs* about 430 µm long or about 5.3 times the corresponding body diameter), comparatively shorter female tail (*c* = 58–76, *c*’ = 0.6–0.8 *vs* *c* = 126, *c*’ = 1.0), and male present (*vs* absent). And from *Aporcelaimoides probulbum* in its shorter neck (663–767 µm, *b* = 3.3–3.7 *vs* 883–1011 µm, *b* = 3.9–5.2), narrower lip region (17–18 *vs* about 21 µm), and comparatively longer tail (*c* = 49–76 *vs* *c* = 75–127).

Moreover, in having short mural odontostyle (11–14 µm at its ventral side) the new species resembles *Aporcelaimoides haguei* (Hunt, 1978), comb. n., but it differs in its smaller general size (*L* = 1.95–2.90 and neck 663–767 µm long *vs* *L* = 4.67–5.42 and neck 1172–1178 µm long), less slender body (*a* = 25–35 *vs* *a* = 52–62), absence (*vs* presence of rows of minute denticles on stomatal wall, indeed a very relevant feature), *pars refringens vaginae* absent (*vs* present), comparatively longer female tail (*c* = 49–76 *vs* *c* = 99–118), and male present (*vs* absent).

#### Type locality and habitat.

Vietnam, Dak Lak province, Chu Yang Sin National Park, where it was collected from soil of a pristine forest in October 2012.

#### Other locality and habitat.

Vietnam, Lam Dong Province, Bidoup-Nui Ba National Park, from soil of a pristine forest, collected in June 2013.

#### Type material.

Female holotype and seven female and nine male paratypes deposited in the nematode collection of the University of Jaén, Spain. One female and one male paratypes deposited in the nematode collection of the Institute of Ecology and Biological Resources, Vietnam.

#### Etymology.

The specific epithet is a compound Latin term referring to the short mural odontostyle that characterizes this species.

#### Remarks.

The two populations examined are very similar in their morphological features and morphometrics, but some minor differences have been also noted, which are herein regarded as intraspecific variation. Thus, the population from Dak Lak province shows a shorter mural odontostyle (ventral side 11–13 *vs* 13–14 µm, dorsal side 13–14 *vs* 14–17 µm, in females) and comparatively longer neck (*b* = 3.3–3.4 *vs* *b* = 3.5–3.7).

### Aporcelaimoides minor sp. n.

Taxon classificationAnimaliaDorylaimidaAporcelaimidae

http://zoobank.org/CD34503A-3436-4A63-A836-7B871E64A60A

Figs 5 , 6 , 7

#### Material examined.

Ten females from three localities, in good state of preservation.

#### Measurements.

See Table 2.

**Table 2.** Morphometrics of *Aporcelaimoides minor* sp. n. and *Aporcelaimoides silvaticum* sp. n. Measurements in µm (except L, in mm), and in the form: mean ± standard deviation (range).

Species		*Aporcelaimoides minor* sp. n.					*Aporcelaimoides silvaticum* sp. n.		
Population		Tay Yen Tu Natural Reserve		Cao Bang Natural Reserve	Chu Yang Sin National Park	Total Range	Cuc Phuong National Park		
		Holotype	Paratypes				Holotype	Paratypes	
Character	n	♀	2♀♀	2♀♀	5♀♀	10♀♀	♀	♀	4♂♂
L		2.12	2.09, 2.24	2.31, 2.17	2.46 ± 0.14 (2.29–2.61)	2.09–2.61	2.60	2.56	2.31 ± 0.24 (2.09–2.58)
a		24	?, 23	29, 26	28.8 ± 2.9 (26–33)	23–33	29	?	30.7 ± 2.1 (28–33)
b		3.3	3.3, 3.6	4.0, 3.5	3.9 ± 0.3 (3.7–4.4)	3.3–4.4	3.6	3.8	3.7 ± 0.4 (3.4–4.2)
c		117	145, 98	142, 146	109.0 ± 17.2 (90–137)	90–146	83	69	79.9 ± 10.8 (67–94)
c’		0.3	0.3, 0.4	0.3 (n=2)	0.5 ± 0.1 (0.3–0.6)	0.3–0.6	0.7	0.7	0.6 ± 0.0 (0.6–0.7)
V		55	56, 52	50, 53	109.0 ± 17.2 (90–137)	48–56	60	58	-
Lip region diam.		19	20, 19	20, 19	19.4 ± 0.7 (19–20)	19–20	17	17	17.2 ± 0.3 (17–18)
Mural odontostyle length at ventral side		14	15 (n=2)	17, 16	14.3 ± 0.6 (14–15)	14–16	12	12	11.3 ± 0.2 (11–12)
Mural odontostyle length at dorsal side		15	16 (n=2)	14, 15	16.0 ± 0.7 (15–17)	15–17	13	13	12.7 ± 0.2 (12–13)
Odontophore length		33	33 (n=2)	33 (n=2)	33.2 ± 0.7 (33–34)	33–34	47	46	40.3 ± 1.3 (39–41)
Guiding ring from ant. end		14	15 (n=2)	14, ?	12.7 ± 1.0 (12–14)	12–15	12	12	10.9 ± 2.0 (9–13)
Neck length		646	632, 630	579, 618	626 ± 28 (592–649)	579–649	720	668	625 ± 40 (597–684)
Pharyngeal expansion length		423	400, 380	331, 382	391 ± 27 (360–420)	331–423	452	400	383 ± 37 (353–436)
Diam. at neck base		81	80, 90	67, 74	83.9 ± 9.7 (69–93)	67–93	87	?	71.9 ± 5.8 (65–79)
at midbody		87	?, 97	80, 83	86.0 ± 9.6 (70–95)	70–97	88	?	75.1 ± 4.8 (69–80)
at anus		57	51 (n=2)	49, 55	51.2 ± 5.4 (46–60)	46–60	48	51	46.3 ± 1.1 (45–48)
Prerectum length		79	76, ?	73, 95	97.0 ± 9.5 (87–106)	73–106	117	114	148.4 ± 5.6 (142–153)
Rectum/cloaca length		47	50 (n=2)	50, 42	52.7 ± 5.3 (47–60)	42–60	55	42	61.5 ± 4.9 (56–66)
Tail length		18	14, 23	16, 15	22.9 ± 3.4 (17–26)	14–26	31	37	29.1 ± 2.4 (27–31)
Spicule length		-	-	-	-	-	-	-	70.4 ± 5.1 (64–75)
Ventromedian supplements		-	-	-	-	-	-	-	2.5 ± 0.6 (2–3)

#### Description.

*Female*. Moderately slender to slender nematodes of medium size, 2.09–2.61 mm long. Body cylindrical, distinctly tapering towards the anterior end, less so towards the posterior one as the caudal region is very short and rounded to truncate. Habitus regularly curved ventrad after fixation, often spiral-shaped. Cuticle three-layered, especially distinguishable at caudal region: thin outer layer bearing fine transverse striation through the entire body, a much thicker intermediate layer with radial striation, and a thin inner layer; thickness 3.0–4.5 µm at anterior region, 4.5–6.5 µm in mid-body and 6.5–9.5 µm on tail. Lateral chord 7–13 µm wide at mid-body, occupying one-tenth to less than one-sixth (9–15%) of mid-body diameter. Two ventral and two dorsal body pores are usually present at level of mural odontostyle-odontophore, their corresponding ducts appearing especially thickened beneath intermediate cuticle layer. Lip region offset by deep constriction, 2.8–3.3 times as wide as high and one-fifth to less than one-third (21–30%) of body diameter at neck base; lips mostly amalgamated, somewhat angular; papillae perceptible, somewhat protruding. Amphid fovea cup-shaped, its opening occupying 8–10 µm or up to one-half (44–50%) of lip region diameter. Cheilostom nearly cylindrical, lacking any differentiation. Mural odontostyle attached subventrally, 6.7–7.7 times as long as wide, 0.7–0.9 times as long as lip region diameter, and 0.54–0.72% of body length; aperture 11–13 µm long or up to six-sevenths (73–84%) its length. Guiding ring simple, somewhat plicate, at 0.6–0.8 lip region diameters from anterior end. Odontophore linear, rod-like, irregular at its base, in lateral view with the ventral side longer that the dorsal one (figure 7A, C), and 2.0–2.4 times the mural odontostyle length. Anterior region of pharynx enlarging very gradually; basal expansion 8–12 times as long as wide, 4.1–5.3 times as long as body diameter and occupying 57–66% of total neck length; gland nuclei obscure in most specimens examined, DN = 54 (n=1) and S_2_N = 92 (n=1). Nerve ring located at 158–177 µm from anterior end or 24–31% of total neck length. Cardia rounded conoid, 11–19 × 14–18 µm; a ring-like structure is present surrounding its junction to pharyngeal base. Genital system didelphic-amphidelphic, with both branches almost equally and well developed, the anterior 174–207 µm long or 7–9% of body length and the posterior 168–220 µm long or 7–9% of body length. Ovaries variably sized, the anterior 93–191 µm, the posterior 84–175 µm long; oocytes arranged first in two or more rows, then in a single row. Oviduct 73–103 µm long or 0.9–1.1 times the corresponding body diameter, and consisting of a slender part with prismatic cells and a weakly developed *pars dilatata*. Oviduct-uterus junction marked by a sphincter. Uterus a short, simple, tube-like structure 44–69 µm long or 0.5–0.9 times the corresponding body diameter, lacking sperm cells inside. Vagina extending inwards 38–51 µm or two-fifths to one-half (43–53%) of body diameter: *pars proximalis* 25–35 × 15–20 µm, with somewhat sigmoid walls and surrounded by weak musculature; *pars refringens* with two small, triangular to drop-shaped pieces measuring 8–10 × 6–8 µm and with a combined width of 14–19 µm; and *pars distalis* short, 3.0–5.5 µm long. Vulva a transverse slit. Prerectum 1.4–2.1, rectum 0.8–1.1 anal body diameters long. Tail very short and rounded to truncate. Caudal pores two pairs, one sublateral, another sub-dorsal.

*Male*. Unknown.

**Figure 5.**
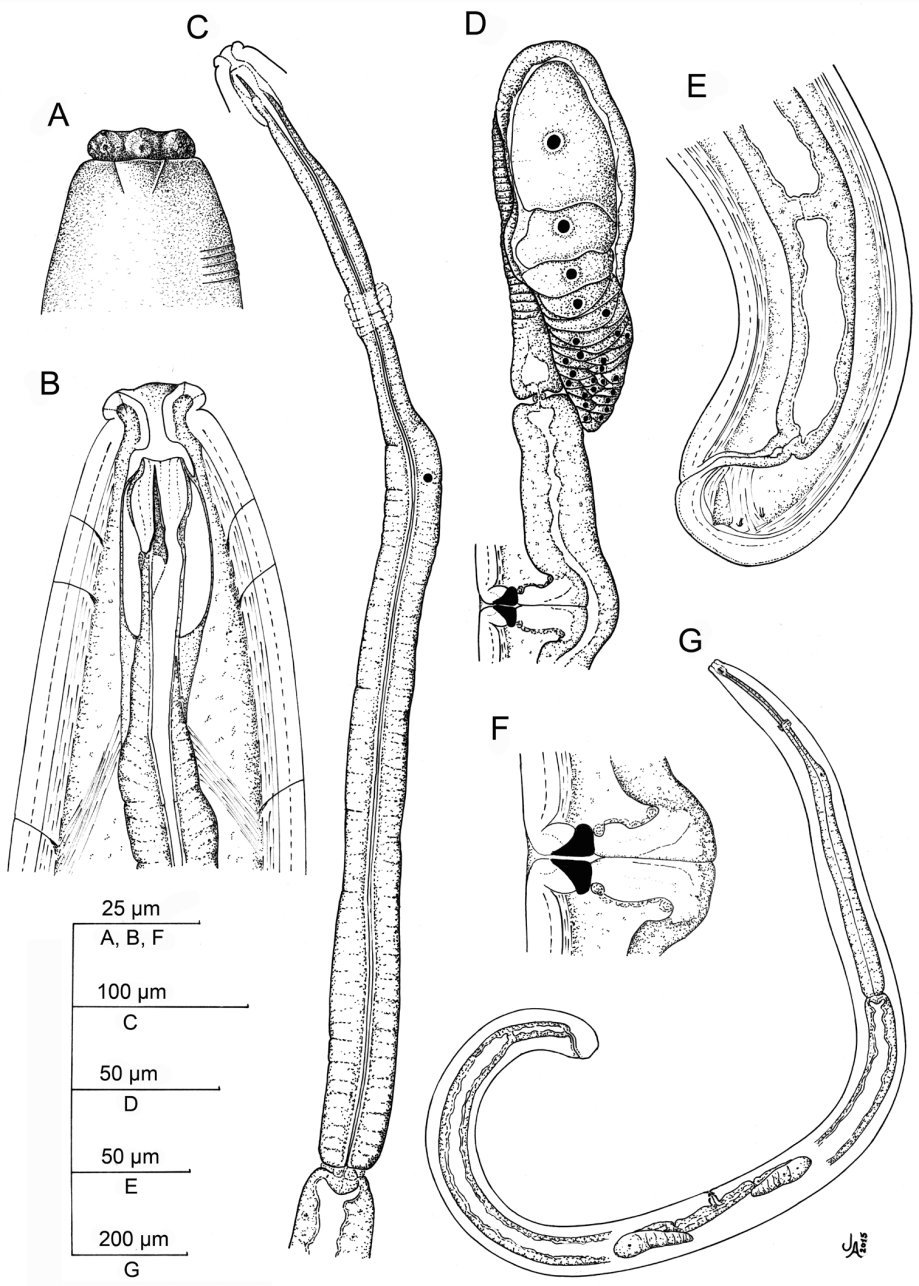
*Aporcelaimoides minor* sp. n. (Female, line drawing). **A** Lip region in surface, lateral view **B** Anterior region in median, lateral view **C** Neck **D** Anterior genital branch and vagina **E** Posterior body region **F** Vagina **G** Entire.

**Figure 6.**
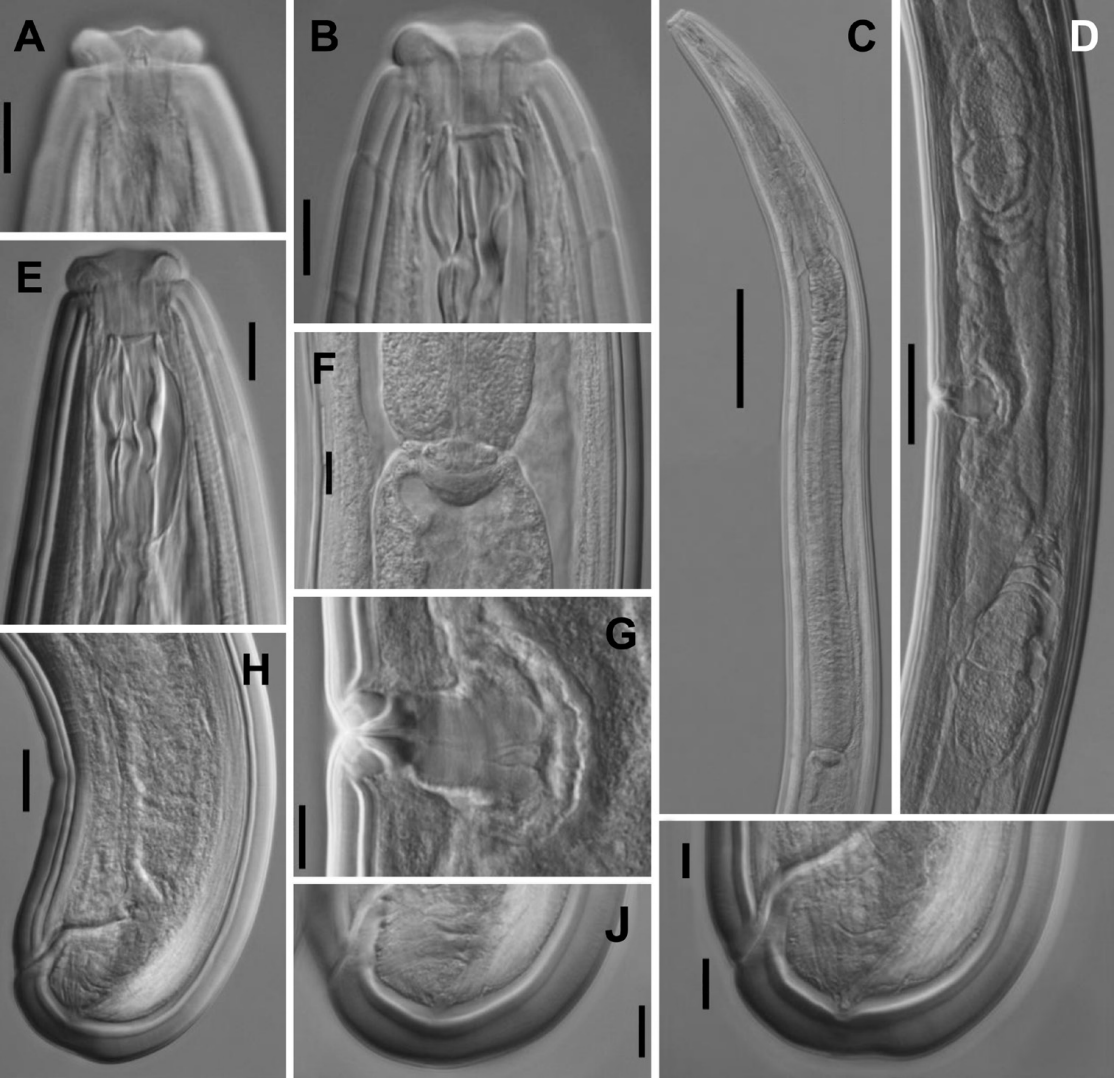
*Aporcelaimoides minor* sp. n. (LM, female, type population). **A** Anterior region in surface, lateral view **B, E** Anterior region in median, lateral view **C** Neck region **D** Genital system **F** Pharyngo-intestinal junction **G** Vagina **H** Posterior body region **I, J** Caudal region. Scale bars: 10 µm (**A, B, E–G, I, J**); 100 µm (**C**); 50 µm (**D**); 20 µm (**H**).

**Figure 7.**
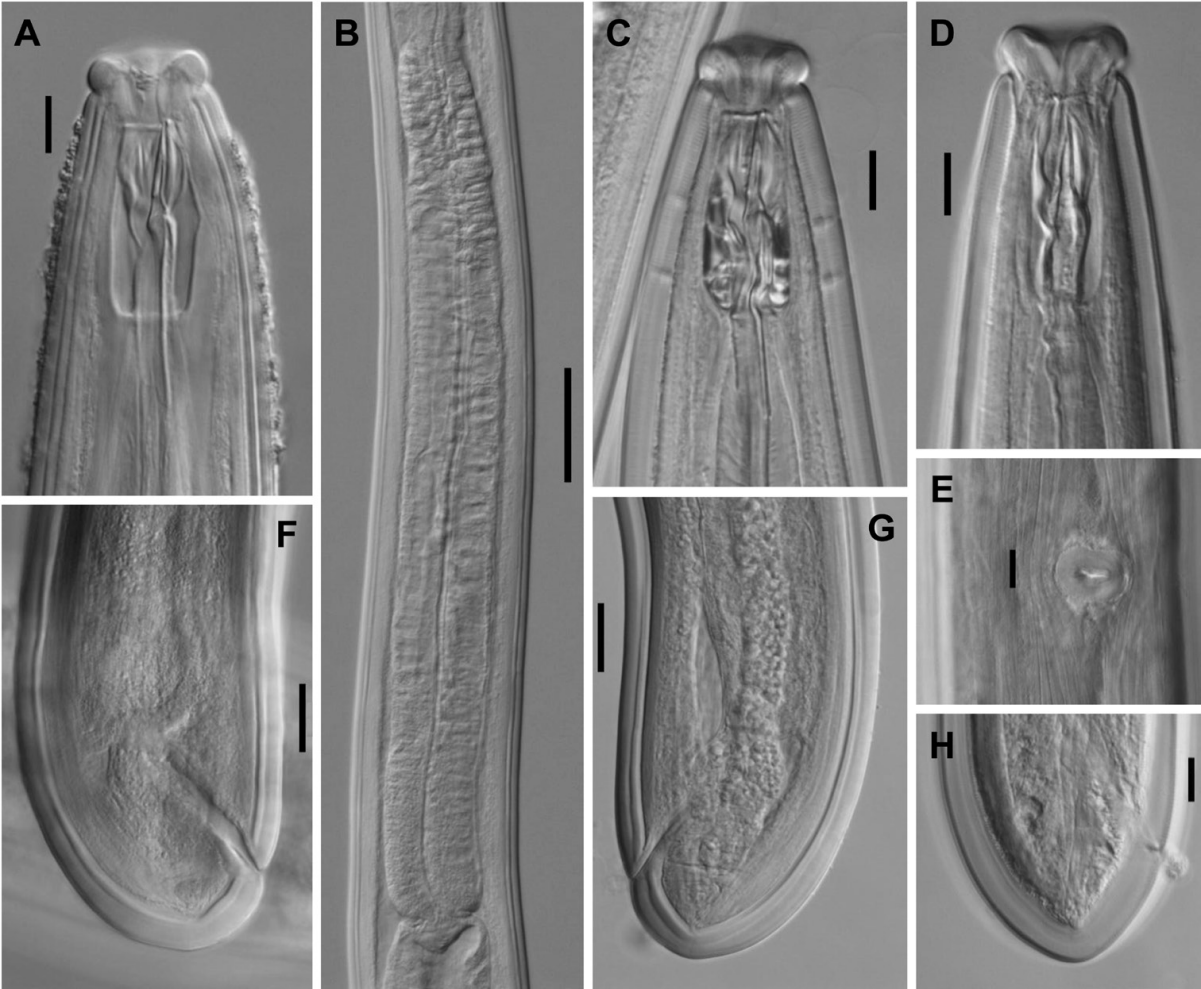
*Aporcelaimoides minor* sp. n. (LM, female, other populations). **A, B, F** (Population from Cao Bang Natural Reserve) **C–E, G, H** (Population from Chu Yang Sin National Park) **A, C** Anterior region in median, lateral view **B** Pharyngeal expansion **D** Anterior region in median, ventral view **E** Vulva in ventral view **F–H** Caudal region. Scale bars: 10 µm (**A, C, D, E, H**); 50 µm (**B**); 20 µm (**F, G**).

#### Diagnosis.

The new species is characterized by its body 2.09–2.61 mm long, lip region offset by deep constriction and 19–20 µm broad, mural odontostyle 14–16 µm long at its ventral side with aperture occupying 73–84% of its length, neck 579–649 µm long, pharyngeal expansion 331–423 µm long or occupying 57–66% of total neck length, uterus a simple tube and 44–69 µm long or less than (0.5–0.9 times) the corresponding body diameter, *pars refringens vaginae* well developed, *V* = 48–56, female tail very short and rounded to truncate (14–26 µm, *c* = 90–146, *c*’ = 0.3–0.6), and male unknown.

#### Relationships.

This species resembles *Aporcelaimoides haguei* comb. n. in having relatively small mural odontostyle (up to 17 µm long) and *pars refringens vaginae* present. It can be, however, easily distinguished from this in its smaller general size (*L* = 2.09–2.61, neck 579–649 µm long *vs* *L* = 4.67–5.42, neck 1112–1178 µm long), less slender body (*a* = 23–33 *vs* *a* = 52–62), the absence (*vs* presence) of rows of minute denticles on stomatal wall), and its much shorter female tail (14–26 µm, *c*’ = 0.3–0.6 *vs* 46–47 µm, *c*’ = 0.7).

Besides, in having short mural odontostyle (11–14 µm at its ventral side) the new species resembles *Aporcelaimoides brevistylum* sp. n. and *Aporcelaimoides californicum*, but it differs from these in its well developed *pars refringens vaginae* (*vs* absent). Moreover, it differs from *Aporcelaimoides brevistylum* sp. n. in its shorter neck (579–649 *vs* 663–767 µm), wider lip region (19–20 *vs* 17–18 µm), smaller mural odontostyle aperture (occupying 73–84% *vs* 62–71% its length), shorter female tail (14–26 µm, *c* = 90–146, *c*’ = 0.3–0.6 *vs* 35–46 µm, *c* = 58–76, *c*’ = 0.6–0.8), and male absent (*vs* present). And from *Aporcelaimoides californicum* in its shorter (*L* = 2.09–2.61 *vs* *L* = 5.53) and less slender (*a* = 23–33 *vs* *a* = 75) body, larger mural odontostyle aperture (occupying 73–84% *vs* one-half of its length), shorter uterus (44–69 µm long or less than one body diameter *vs* about 430 µm long or about 5.3 times the corresponding body diameter), and shorter female tail (14–26 µm, *c*’ = 0.3–0.6 *vs* 44 µm, *c*’ = 1.0).

#### Type locality and habitat.

Vietnam, Bac Giang Province, Tay Yen Tu Natural Reserve, collected from soil in a pristine tropical forest, in July 2008.

#### Other localities and habitats.

Vietnam, Cao Bang Province, Cao Bang Natural Reserve (GPS coordinates: 22°34'07"N and 105°52'34"), in a tropical evergreen forest soil in association with *Dipterocarpus* sp. and *Cinnamomum* sp., collected in 2013. Dak Lak province, Chu Yang Sin National Park, in October 2012.

#### Type material.

Female holotype and one female paratype deposited in the nematode collection of the University of Jaen, Spain. One female paratype deposited in the nematode collection of the Institute of Ecology and Biological Resources, Vietnam.

#### Etymology.

The specific epithet means ‘small’ and refers to the comparatively small general size of the new species.

#### Remarks.

In spite of it was collected from three localities, the material examined is very similar in its main morphological features and morphometrics. Nevertheless, some differences have been also observed, especially affecting the female tail shape as some specimens show a short and rounded-conoid caudal region whereas it becomes extremely short and truncate in other individuals.

### Aporcelaimoides silvaticum sp. n.

Taxon classificationAnimaliaDorylaimidaAporcelaimidae

http://zoobank.org/C8EAC3A3-EB59-485C-86DC-1471D682731A

Figs 4F–I , 8 , 9

#### Material examined.

Two females and four males, in variable state of preservation.

#### Measurements.

See Table 2.

#### Description.

*Adult*. Moderately slender to slender nematodes of medium size, 2.09–2.60 mm long. Body cylindrical, distinctly tapering towards the anterior end, less so towards the posterior one because the caudal region is rounded conoid. Habitus regularly curved ventrad after fixation, to a more or less open C, occasionally more curved at posterior body region, spiral-shaped in only one male specimen. Cuticle three-layered, especially visible distinct at caudal region, consisting of thin outer layer bearing fine transverse striation through the entire body, a much thicker intermediate layer with radial striation, and a thin inner layer; thickness 2.5–4.0 µm at anterior region, 5–6 µm in mid-body and 8–10 µm on tail. Lateral chord 7–12 µm wide at mid-body, occupying one-tenth to one-eighth (10–12%) of mid-body diameter. Three ventral and three dorsal body pores are usually present at level of odontostyle-odontophore, their corresponding ducts appearing especially thickened beneath inner cuticle layer. Lip region visibly narrower than adjacent body, offset by depression, 2.2–2.6 times as wide as high and one-fifth to one-fourth (19–27%) of body diameter at neck base; lips (under SEM) amalgamated; papillae button-like, the inner labial ones rather close the margin of oral field and surrounded by one or two ring-like annuli, whereas the outer labial ones are surrouned by only one annulus and the cephalic ones lack a such differentiation; oral aperture a dorsoventral, nearly hexagonal orifice, the lip region hence showing a biradial symmetry. Amphid fovea cup-shaped, its opening at level of cephalic depression and occupying 12–13 µm or up to three-fourths (72–75%) of lip region diameter. Cheilostom nearly cylindrical, lacking any differentiation. Mural odontostyle attached subventrally and comparatively short, 4.5–4.9 times as long as wide, 0.6–0.7 times as long as lip region diameter, and 0.44–0.54% of body length; aperture 7–8 µm long or up to two-thirds (60–66%) its length. Guiding ring simple, somewhat plicate, at 0.5–0.7 lip region diameters from anterior end. Odontophore linear, rod-like, 3.8, 4.1 (3.5, 3.7 in males, n=2) times the odontostyle length. Anterior region of pharynx enlarging very gradually; basal expansion 8.3–11.0 times as long as wide, 5.0–5.8 times as long as body diameter, and occupying 58–64% of total neck length; gland nuclei obscure in all the specimens examined. Nerve ring located at 139–169 µm from anterior end or 23–26% of total neck length. Cardia rounded conoid, 10–16 × 12–18 µm; a ring-like structure is present surrounding its junction to pharyngeal base. Tail short, rounded to rounded conoid, its inner core bearing a finger-like projection at tail end. Caudal pores two pairs, one lateral, another sub-lateral.

*Female*. Genital system didelphic-amphidelphic, with both branches almost equally and well developed, the anterior 246, 387 µm long or 10, 15% of body length and the posterior 301 µm long or 12% (n=1) of body length. Ovaries moderately sized, usually not surpassing the sphincter level, the anterior 82, 119 µm, the posterior 66, 103 µm long; oocytes arranged first in two or more rows, then in a single row. Oviduct 99–107 µm long or 1.2, 1.3 times the corresponding body diameter, and consisting of a slender part with prismatic cells and a moderately developed *pars dilatata* with visible lumen but no sperm cell. Oviduct-uterus junction marked by a sphincter. Uterus a short, simple, tube-like structure 128–243 µm long or 2.1, 2.8 times the corresponding body diameter, one female containing abundant sperm cells inside. Vagina extending inwards 31, 32 µm or about three-eighths (36%, n=1) of body diameter: *pars proximalis* 23, 24 × 25, 26 µm, with somewhat sigmoid walls and surrounded by weak musculature; *pars refringens* with two small, triangular to drop-shaped pieces measuring 5 × 4, 5 µm and with a combined width of 9, 10 µm; and *pars distalis* 1.0, 1.5 µm long. Vulva a post-equatorial transverse slit. Prerectum 2.3, 2.5, rectum 0.8, 1.2 anal body diameters long.

*Male*. Genital system diorchic, with opposite testes. In addition to the ad-cloacal pair, situated at 16–19 µm from cloacal aperture, there is a series of two or three widely spaced (22–42 µm apart) ventromedian supplements, the posteriormost of which lying out the range of spicules, but very close to the spicules end, being situated at 42–63 µm from ad-cloacal pair. Spicules relatively robust, 3.5–4.6 times its maximum width, 1.3–1.6 times the body diameter at level of the cloacal aperture: dorsal contour regularly convex, ventral contour bearing weak hump and hollow; curvature 140–143º; head occupying 8–10% of spicule total length, with both contours nearly straight, and its dorsal side longer than the ventral one; median piece 6.5–8.3 times as long as wide, occupying 45–54% of spicule maximum width, reaching the posterior tip; posterior end 5–6 µm wide. Lateral guiding pieces 19–23 µm long, 6.4–7.8 times as long as wide. Prerectum 3.0–3.3, cloaca 1.2–1.4 times the corresponding body width long.

**Figure 8.**
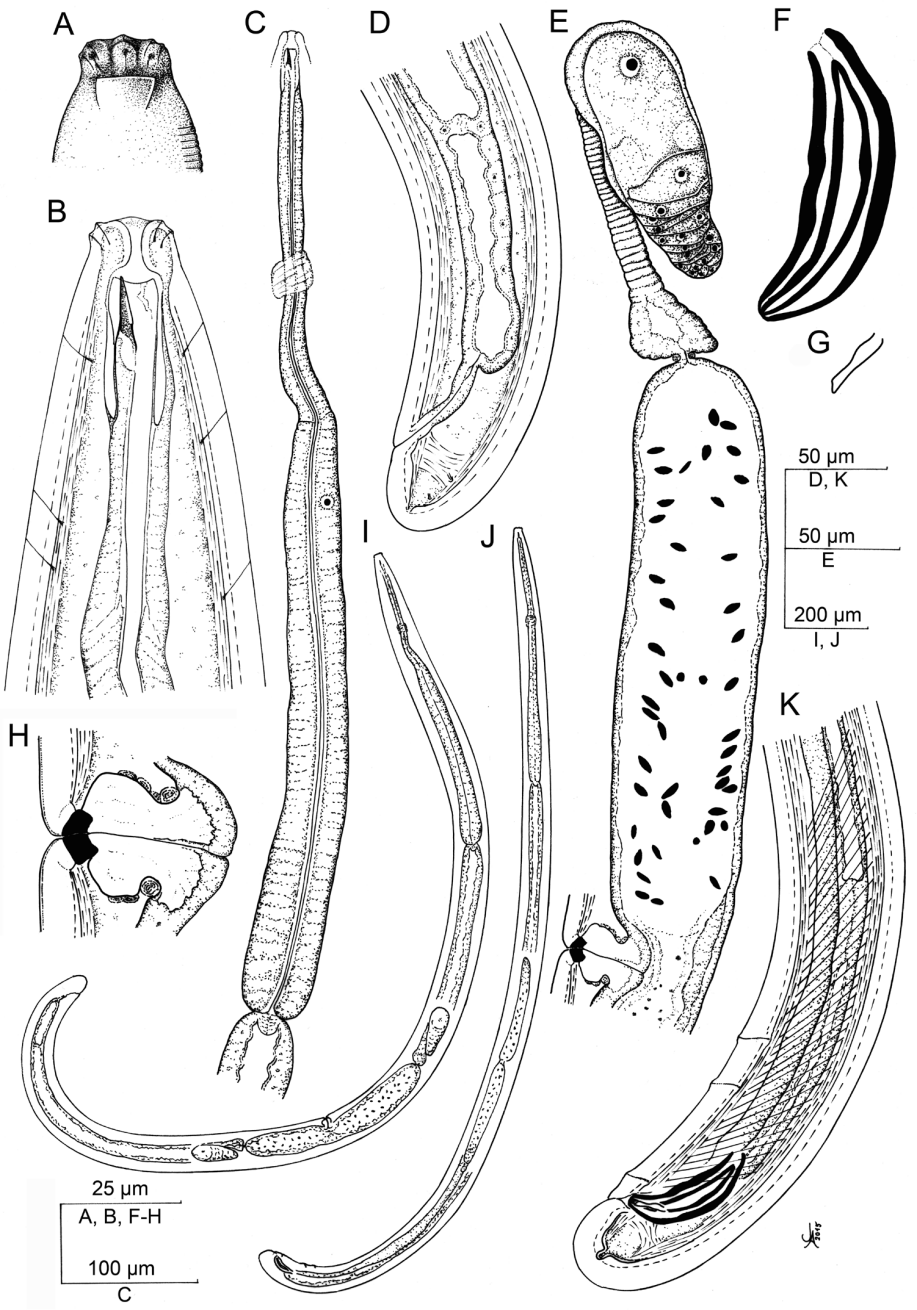
*Aporcelaimoides silvaticum* sp. n. (Line drawing). **A** Lip region in surface, lateral view **B** Anterior region in lateral, median view **C** Neck **D** Female, posterior body region **E** Female, anterior genital branch and vagina **F** Spicule **G** Lateral guiding piece **H** Vagina **I** Female, entire **J** Male, entire **K** Male, posterior body region.

**Figure 9.**
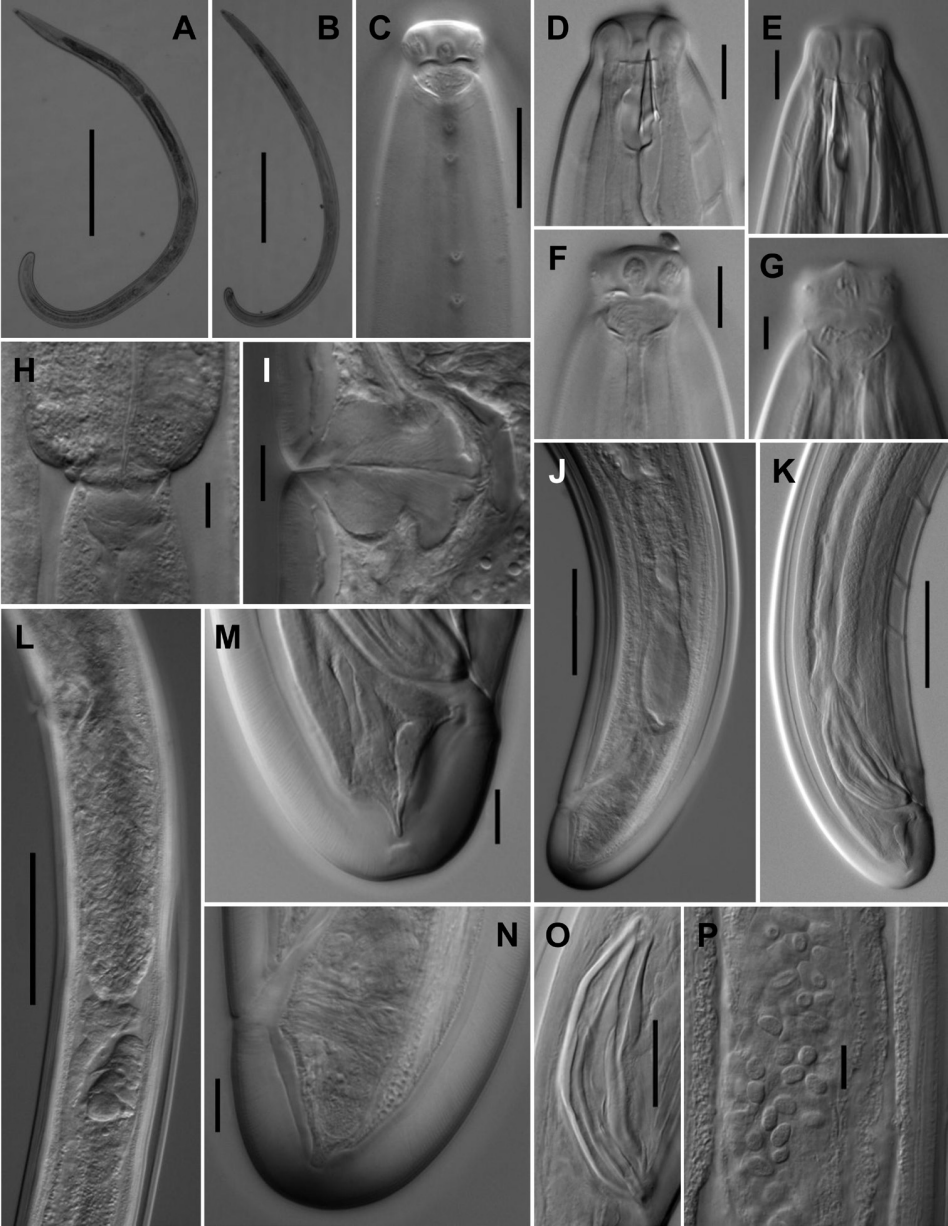
*Aporcelaimoides silvaticum* sp. n. (LM). **A** Female, entire **B** Male, entire **C** Anterior region in surface, lateral view showing four lateral pores **D, E** Anterior region in median, lateral view **F, G** Anterior region in surface, lateral view showing the amphid fovea **H** Pharyngo-intestinal junction **I** Vagina **J** Female, posterior body region **K** Male, posterior body region **L** Female, posterior genital branch **M** Male, caudal region **N** Female, caudal region **O** Spicules **P** Sperm cells. Scale bars: 500 µm (**A, B**); 20 µm (**C**); 10 µm (**D–F, H, I, M–P**); 5 µm (**G**); = 50 µm (**J–L**).

#### Diagnosis.

The new species is characterized by its body 2.09–2.60 mm long, lip region offset by depression and 17–18 µm broad, mural odontostyle 11–12 µm long at its ventral side with aperture occupying 60–66% of its length, neck 597–720 µm long, pharyngeal expansion 353–452 µm long or occupying 58–64% of total neck length, uterus a simple tube and 128–243 µm long or 2.1–2.8 times the corresponding body diameter, *pars refringens vaginae* well developed, *V* = 58–60, female tail short and rounded to rounded conoid (31–37 µm, *c* = 69–83, *c*’ = 0.7), male tail similar to that of female (27–31 µm, *c* = 67–94, *c*’ = 0.6–0.7), spicules 64–75 µm long, and two or three widely spaced ventromedian supplements bearing hiatus.

#### Relationships.

The new species differs from its relatives by its lip region offset by depression (*vs* constriction). Besides, in having *pars refringens vaginae* and short mural odontostyle (11–12 µm long at its ventral side), *Aporcelaimoides silvaticum* sp. n. is morphologically similar to *Aporcelaimoides haguei* comb. n. and *Aporcelaimoides minor* sp. n., but it can be distinguished from them in its narrower lip region (17–18 *vs* equal or 19 µm or more) and more posterior vulva (*V* = 58–60 *vs* *V* up to 57). It also differs from *Aporcelaimoides haguei* comb. n. in its smaller general size (*L* = 2.09–2.60, neck 597–720 µm long *vs* *L* = 4.67–5.42, neck 1112–1178 µm long), less slender body (*a* = 28–33 *vs* *a* = 52–62), absence (*vs* presence) of rows of minute denticles on stomatal wall), shorter female tail (31–37 *vs* 46–47 µm), and male present (*vs* absent). And from *Aporcelaimoides minor* sp. n. in its shorter mural odontostyle (11–12 *vs* 14–16 µm at its ventral side) with smaller aperture (occupying 60–66% *vs* 73–84% of its length), longer female tail (27–31 µm, *c* = 67–94, *c*’ = 0.6–0.7 *vs* 14–26 µm, *c* = 90–146, *c*’ = 0.3–0.6), and male present (*vs* absent). Finally, the new species also resembles *Aporcelaimoides brevistylum* sp. n. in having short mural odontostyle, but it differs from this in its well developed (*vs* absent) *pars refringens vaginae* and higher number of ventromedian supplements (two or three *vs* one).

#### Type locality and habitat.

Northern Vietnam, Cuc Phuong National Park, where the new species was collected from soil of a pristine tropical forest in 2009.

#### Type material.

Female holotype and one female and three male paratypes, deposited in the nematode collection of the University of Jaen, Spain. One male paratype deposited in the nematode collection of the Institute of Ecology and Biological Resources, Vietnam.

#### Etymology.

The specific epithet is a Latin term meaning ‘from the jungle’, and refers to the habitat where the species dwells.

### On the taxonomy of *Aporcelaimoides*

As mentioned in the introductory section, the identity of *Aporcelaimoides* has been matter of some controversy. In his original description of this genus, Heyns (1965) highlighted the differences between it and *Sectonema*, mainly based on the nature of the stomatal protrusible structure, “a dorylaimid spear which is set ventrally … similar in position to the mural tooth of *Sectonema*”, putting hence emphasis on the existence of a large dorsal aperture in the protrusible structure of *Aporcelaimoides* and the absence of a such aperture in the mural tooth of *Sectonema*. Heyns (*op. cit.*) also noted that “the basal part of the spear (is) much narrower than the lumen of the pharynx (stoma)” [text between brackets incorporated by the authors]. Subsequent contributions by Andrássy (1976) and Jairajpuri and Ahmad (1992) assumed Heyns’ point of view. Siddiqi (1995), however, stated (p. 99) that “…Since there is a great variation in the mural tooth of *Sectonema* (see Siddiqi 1984), there is no justification for holding *Aporcelaimoides* as a valid genus”. And, later, Andrássy (2009) followed Siddiqi’s opinion.

Very recently, Peña-Santiago and Álvarez-Ortega (2014a) redescribed *Sectonema ventrale* Thorne, 1930, the type species of *Sectonema*, and conclude that (p. 1103) “the protrusible structure of *Sectonema*, as observed in its type species, is not a typical mural tooth as seen in nygolaims, but a reduced odontostyle with its base occupying most (if not whole) the stomatal lumen”. It means that mural odontostyle of *Aporcelaimoides* significantly differs from the reduced odontostyle of *Sectonema*. Thus, Siddiqi’s (1995) action might be not well supported as there are morphological arguments to separate both genera and to restore *Aporcelaimoides* as valid genus. Unfortunately, there is no molecular information of the latter, which would be especially useful to confirm the morphological data.

A revised diagnosis of *Aporcelaimoides* as well an updated list of its species, three of them transferred from *Sectonema*, and a key to their identification are given in the following. Besides, a compendium of their main morphometrics is presented in Table 3.

**Table 3.** Main morphometrics and distribution data of species belonging to the genus *Aporcelaimoides* Heyns, 1965 (Measurements in µm, except L, in mm).

	Character** Species	n	L	a	b	c	c’	V	Lrd	Mural Odont. Vent.	Mural Odont. Dors.	Neck	Ph. exp.	Tail	Spicul.	Ve. Sup.	Geog. Dis.	Reference
1	***amazonicum*** comb. n.	♀	3.34	43	3.4	88	0.8	53	27	25	30	991*	62%	38*	-	-	Brazil	Sidiqqi 1995
		♂	3.30	53	4.5	81	1.0	-	21*	?	23	733*	?	41*	78	3		
2	***brevistylum*** sp. n.	12♀♀	2.33–2.90	25–33	3.3–3.5	58–76	0.6–0.8	55–63	17–18	11–14	13–17	695–767	58–66%	35–46	-	-	Vietnam	Present paper
		14♂♂	1.95–2.60	27–35	3.3–3.7	49–69	0.6–0.8	-	17–18	11–12	13–14	663–685	58–60%	34–42	67–86	1		
3	***californicum***	♀	5.53	75	7.6	126	1.0	51	19*	16*	17	728*	?	44*	-	-	California-USA	Heyns 1965
4	***haguei*** comb. n.	2♀♀	4.67–5.42	52–62	4.2–4.6	99–118	0.7	56–57	24*	?	16	1112–1178*	71%*	46–47*	-	-	St. Lucia	Hunt 1978
5	***minor*** sp. n.	10♀♀	2.09–2.61	23–33	3.3–4.4	90–146	0.3–0.6	48–56	19–20	14–16	15–17	579–649	57–66%	14–26	-	-	Vietnam	Present paper
6	***moderatum*** comb. n.	2♀♀	4.34–5.66	37–38	3.9–4.2	82–105	0.7	55–59	25*	?	25–26.5	1113–1348*	?	53–54*	-	-	Cameroon	Siddiqi 1995
7	***probulbum***	11♀♀	3.35–4.75	41–55	3.9–5.2	75–127	0.6*	50–60	21*	15.5*	17–20	883*	64–67%	33*	-	-	South Africa	Heyns 1965
		11♂♂	3.62–4.66	49–63	4.0–4.9	78–118	0.8*	-	?	?	?	991*	?	44*	75–90	0–4		
		2♀♀	4.35–4.70	51–55	5.3–5.4	117–150	0.5–0.7	53–54	?	?	18	813–882	?	29–40	-	-	India	Khan et al. 1989
8	***silvaticum*** sp. n.	2♀♀	2.60, 2.56	29, ?	3.6, 3.8	83, 69	0.7	60, 58	17	12	13	720, 668	63, 60%	31, 37	-	-	Vietnam	Present paper
		4♂♂	2.09–2.58	28–33	3.4–4.2	67–94	0.6–0.7	-	17–18	11–12	12–13	597–684	58–64%	27–31	64–75	2–3		

* Calculated from original description.

** Abbreviations for columns: Lrd: Lip region diameter. Mural Odont. Vent: Mural odontostyle length at ventral side. Mural Odont. Dors.: Mural odontostyle length at dorsal side. Ph.exp.: Pharyngeal expansion length. Spicul.: Spicule length. Ve.sup.: Number of ventromedian supplements. Geog.dis.: Geographical distribution. ? This information is not available in the corresponding description.

### Diagnosis (emended)

Aporcelaimidae. Slender to very slender nematodes (*a* = 23–75) of medium to large size, 1.95–5.66 mm long. Cuticle three-layered, especially obvious at caudal region, with the intermediate layer more refringent and thicker than the outer and the inner ones. Oral aperture a dorso-ventral, nearly hexagonal slit. Lip region offset by a more or less distinct constriction, but by depression in *Aporcelaimoides silvaticum* sp. n. Mural odontostyle attached subventrally, comparatively short and with wide aperture, often occupying more than one-half its length. Guiding ring simple and plicate. Odontophore rod-like. Pharynx enlarging gradually, with basal expansion occupying three-fifths to two-thirds of total neck length. Female genital system didelphic-amphidelphic; *pars refringens vaginae* present or absent; and vulva a transverse slit. Tail similar in both sexes, short, rounded conoid, rounded or truncate. Spicules dorylaimoid, well developed. Ventromedian supplements in low number (0–4), widely separated, always with pre-cloacal space (hiatus).

### Relationships

As mentioned, *Aporcelaimoides* is morphologically very similar to *Sectonema*, from which it differs in the nature of the stomatal protrusible structure. It can be easily distinguished from the typical species of *Sectonema*, for instance *Sectonema ventrale* — the type species of the genus, recently re-described by Peña-Santiago and Álvarez-Ortega (2014a) — in having a mural odontostyle attached to the ventral side of stoma (*vs* a reduced axial odontostyle), much narrower than (*vs* occupying the whole) stomatal lumen, with (in lateral view) its dorsal and ventral sides parallel and distinctly perceptible (*vs* dorsal side nearly lost) and a perceptible dorsal aperture often occupying more than half of its total length (*vs* nearly the total odontostyle length). Besides, *Aporcelaimoides* compares to other atypical species of *Sectonema*, for instance *Sectonema demani* Altherr, 1965 (see recent description by Peña-Santiago and Álvarez-Ortega 2014b) and *Sectonema septentrionale* Peña-Santiago & Álvarez-Ortega, 2015, which are characterized by having a mural tooth (a protrusible structure lacking a distinct aperture, resembling that found in nygolaims) with asymmetrical sides as (in lateral view) the dorsal side is visibly sigmoid and distinctly longer than the ventral one.

### List of species

#### Type species

*Aporcelaimoides probulbum* Heyns, 1965

= *Sectonema probulbum* (Heyns, 1965) Siddiqi, 1995

#### Other valid species

*Aporcelaimoides amazonicum* (Siddiqi, 1995), comb. n.

= *Sectonema amazonicum* Siddiqi, 1995

*Aporcelaimoides brevistylum* sp. n.

*Aporcelaimoides californicum* Heyns, 1965

= *Sectonema californicum* (Heyns, 1965) Siddiqi, 1995

*Aporcelaimoides haguei* (Hunt, 1978), comb. n.

= *Sectonema haguei* (Hunt, 1978) Andrássy, 2009

*Nygolaimium haguei* Hunt, 1978

*Aporcelaimoides minor* sp. n.

*Aporcelaimoides moderatum* (Siddiqi, 1995), comb. n.

= *Sectonema moderatum* Siddiqi, 1995

*Aporcelaimoides silvaticum* sp. n.

### Remarks on some species

*Aporcelaimoides amazonicum*: The nature of the stomatal protrusible structure, a mural odontostyle, supports its inclusion in *Aporcelaimoides* rather than in *Sectonema*.

*Aporcelaimoides haguei*: Andrássy (2009) transferred this species to *Sectonema* from *Nygolaimium*, but the mural odontostyle that characterized this species justifies its transference to *Aporcelaimoides*.

*Aporcelaimoides moderatum*: The general morphology of this species, very especially that of the stomatal protrusible structure, fits the updated concept of *Aporcelaimoides* and justifies its transference to this genus.

### Key to identification of *Aporcelaimoides* species

**Table:** 

1	Lip region nearly continuous with the adjacent body	***silvaticum* sp. n.**
–	Lip region offset by constriction	**2**
2	*Pars refringens vaginae* absent	**3**
–	*Pars refringens vaginae* present	**5**
3	Larger (body 5.53 mm long) and more slender (*a* = 75) nematodes; neck comparatively shorter (*b* = 7.6); mural odontostyle aperture occupying one-half its length; male absent	***californicum***
–	Smaller (body up to 4.75 mm long) and less slender (*a* up to 63) nematodes; neck comparatively longer (*b* up to 5.4); mural odontostyle aperture occupying 62–71% its length; male present	**4**
4	Body 1.95–2.90 mm long and less slender (*a* = 25–35); shorter neck (663–767 µm long, *b* = 3.3–3.7); lip region 17–18 µm wide; comparatively longer tail (*c* = 49–76)	***brevistylum* sp. n.**
–	Body 3.35–4.75 mm long and more slender (*a* = 41–63); longer neck (883–1011 µm long, *b* = 3.9–5.2); lip region about 21 µm wide; comparatively shorter tail (*c* = 75–127)	***probulbum***
5	Mural odontostyle 23 µm long or more	**6**
–	Mural odontostyle up to 17 µm long	**7**
6	Smaller general size (body 3.30–3.34 mm, neck 733–991 µm long); more slender body (*a* = 43–53); mural odontostyle aperture occupying 80–85% its length; vulva more anterior (*V* = 53); female tail 38 µm long; male present	***amazonicum* comb. n.**
–	Larger general size (body 4.34–5.66 mm, neck 1113–1348 µm long); less slender body (*a* = 37–38); mural odontostyle aperture occupying 66–75% its length; vulva more posterior (*V* = 55–59); female tail 53–54 µm long; male absent	***moderatum* comb. n.**
7	Smaller general size (body 2.09–2.61 mm, neck 579–649 µm long); more obese body (*a* = 23–33); stomatal walls lacking rows of minute denticles; mural odontostyle occupying 73–84% its length; shorter tail (14–26 µm long, *c*’ = 0.3–0.6)	***minor* sp. n.**
–	Larger general size (body 4.67–5.42 mm, neck 1112–1178 µm long); more slender body (*a* = 52–62); stomatal walls bearing rows of minute denticles; mural odontostyle aperture occupying 69% its length; longer tail (46–47 µm long, *c*’ = 0.7)	***haguei* comb. n.**

## Supplementary Material

XML Treatment for Aporcelaimoides brevistylum

XML Treatment for Aporcelaimoides minor

XML Treatment for Aporcelaimoides silvaticum
